# A Combined Metabolomic and Proteomic Study Revealed the Difference in Metabolite and Protein Expression Profiles in Ruminal Tissue From Goats Fed Hay or High-Grain Diets

**DOI:** 10.3389/fphys.2019.00066

**Published:** 2019-02-08

**Authors:** Changzheng Guo, Daming Sun, Xinfeng Wang, Shengyong Mao

**Affiliations:** ^1^Jiangsu Key Laboratory of Gastrointestinal Nutrition and Animal Health, Laboratory of Gastrointestinal Microbiology, National Experimental Teaching Demonstration Center of Animal Science, National Center for International Research on Animal Gut Nutrition, Joint International Research Laboratory of Animal Health and Food Safety, College of Animal Science and Technology, Nanjing Agricultural University, Nanjing, China; ^2^College of Animal Science and Technology, Shihezi University, Shihezi, China

**Keywords:** goat, metabolomics, proteomic, rumen epithelium, high grain feeding

## Abstract

Currently, knowledge about the impact of high-grain (HG) feeding on metabolite and protein expression profiles in ruminal tissue is limited. In this study, a combination of proteomic and metabolomic approaches was applied to evaluate metabolic and proteomic changes of the rumen epithelium in goats fed a hay diet (Hay) or HG diet. At the metabolome level, results from principal component analysis (PCA) and PLS-DA revealed clear differences in the biochemical composition of ruminal tissue of the control (Hay) and the grain-fed groups, demonstrating the evident impact of HG feeding on metabolite profile of ruminal epithelial tissues. As compared with the Hay group, HG feeding increased the levels of eight metabolites and decreased the concentrations of seven metabolites in ruminal epithelial tissues. HG feeding mainly altered starch and sucrose metabolism, purine metabolism, glyoxylate and dicarboxylate metabolism, glycerolipid metabolism, pyruvate metabolism, glycolysis or gluconeogenesis, galactose metabolism, glycine, serine and threonine metabolism, and arginine and proline metabolism in ruminal epithelium. At the proteome level, 35 differentially expressed proteins were found in the rumen epithelium between the Hay and HG groups, with 12 upregulated and 23 downregulated proteins. The downregulated proteins were related to fatty acid metabolism, carbohydrate metabolic processes and nucleoside metabolic processes, while most of upregulated proteins were involved in oxidative stress and detoxification. In general, our findings revealed that HG feeding resulted in differential proteomic and metabolomic profiles in the rumen epithelia of goats, which may contribute to better understanding how rumen epithelium adapt to HG feeding.

## Introduction

In modern intensive animal production, feeding high-grain (HG) diets to feedlot goats has become a common practice to meet the energy requirements for the maintenance of the production performance (Penner et al., 2011). However, although HG feeding can increase energy available to the animal, there is a risk of developing acidotic ruminal epithelial damage, thus affecting nutrient absorption. The rumen epithelium is well known to play an important role in maintaining the host’s energy balance and health (Gabel et al., 2002). Previous studies revealed that the short chain volatile fatty acids (SCVFAs) absorbed by the rumen epithelium can meet up to 70% of the energy requirement of ruminants (Bergman, 1990; Penner et al., 2011). In addition, rumen epithelial cells also are the first line of defense against hostile rumen conditions such as acidic pH, high osmotic pressure, and harmful microbial-derived metabolites, such as lipopolysaccharide (LPS) and histamine (Penner et al., 2011). In recent years, the effect of HG feeding on rumen epithelial morphology (Bannink et al., 2008) and physiological functions (Uppal et al., 2003) have been widely investigated, and the results revealed that HG feeding increased length and surface of rumen papilla (Shen et al., 2004) and also impact rumen epithelial absorption, barrier, and immune function (Liu et al., 2013). Undoubtedly, these findings improved our understanding of the adaptative mechanism of the ruminal epithelium in response to HG diet feeding. However, to our knowledge, previous studies are only focused on one level (gene, metabolite or morphology) to investigate the response of rumen epithelial function to HG feeding, which is limited to understand the global change in physiological functions of rumen epithelium during HG feeding.

In recent years, omics technologies have been widely used in understanding the biological mechanism of ruminant (Ametaj et al., 2010; Bondzio et al., 2011), and this also makes it possible to investigate the changes in the physiological functions of rumen epithelium to HG diet comprehensively, therefore it will be beneficial for modulating the animal performance and minimizing the negative effect of HG feeding on rumen health. Up to now, only a few studies have attempted to investigate the effect of HG feeding on rumen epithelium based on the omics technologies. For example, Bondzio et al. (2011) used two dimensional-differential in gel electrophoresis (2D-DIGE) based proteome analysis methods and identified differentially expressed proteins related to morphological alterations of the ruminal epithelium adapting to HG feeding. However, 2D-DIGE does not allow for the detection of regulatory proteins (Gerber et al., 2003). As compared with the 2D-DIGE proteomics methods, label-free liquid chromatography–tandem mass spectrometry (LC–MS/MS) approach is reported to be particularly effective for large-scale protein identification (Lin et al., 2003). Thus, a label-free based proteomic method would provide more information on the alternation in function of the rumen epithelial during HG feeding.

In addition to its role in SCVFA absorption and as a selective barrier, the ruminal epithelium also plays an important role in the metabolism of SCVFA (Bannink et al., 2016). Until now, the effect of different dietary strategy on metabolic function of ruminal epithelial tissue has rarely been studied, and the knowledge of how the epithelial tissue responds to an HG diet feeding is very limited. Accordingly, it is of great interest to gain further insight into how HG diet feeding strategies affect metabolite profiles and function of rumen epithelial tissue. Metabolomics, one of omics technology, has been reported to be a useful approach to characterize the global metabolites of rumen fluid in goats and dairy cows (Ametaj et al., 2010; Mao et al., 2016), and thereby it will be possible to enable a more quantitative characterization of the biochemical composition of this tissue and thereby provide a method to investigate the metabolic activity of the ruminal epithelial tissue. However, until now, few studies have been conducted on investigating the changes in metabolic characterization of ruminal epithelium during HG feeding.

In the present study, we hypothesized that goats fed HG diet or hay diet had differences in profile of metabolomics and proteomics. Therefore, a gas chromatography–mass spectrometry (GC–MS) based metabolomics method and a label-free LC-MS/MS proteomics method was used to characterize the proteomic and metabolic response of rumen papillae to HG diets.

## Materials and Methods

### Animals, Diets, and Experimental Design

The experimental design and procedures for this study were approved by the Animal Care and Use Committee of Nanjing Agricultural University following the requirements of the Regulations for the Administration of Affairs Concerning Experimental Animals (The State Science and Technology Commission of P. R. China, 1988). The current study is a continuation of previous research, where the effect of HG diets on the function and health of the rumen by traditional research methods was investigated (Liu et al., 2013). In the current study, we mainly paid attention to the effect of HG feeding on metabolic and proteomic profiles of rumen epithelium in goats. The experiment design and treatments are described in detail (Liu et al., 2013). Briefly, a total of 10 rumen-cannulated male goats of 2–3 years old were used in the experiment. A pure hay diet was provided for all the goats *ad libitum* for 5 weeks before the experiment treatment, and then, all animals were placed in individual pens (1.2 × 1.2 m) and randomly allocated into two groups. The body weights of the goats between the two groups had no significant difference (29.8 ± 0.86 vs. 30.0 ± 1.05 kg; *P* = 0.886) at the beginning of the feeding trial. One was the Hay group that was fed a hay diet (Hay; 81% *Leymus chinensis*, 15% lucerne, 0.5% CaCO_3_, 0.8% NaCl, 1.7% CaHPO_4_, and 1% mineral and vitamin supplement; 101 g crude protein/kg DM, and 570 g neutral-detergent fiber/kg DM; *n* = 5), and the other was the HG group that was fed an HG diet (HG; 30% *Leymus chinensis*, 45% maize meal, 20% wheat meal, 1.1% soybean meal, 0.95% CaCO_3_, 0.65% NaCl, 1.2% CaHPO_4_, 1% mineral and vitamin supplement, and 0.1% NaHCO_3_; 101 g crude protein/kg DM, 252 g neutral-detergent fiber/kg DM, and 582 g starch/kg DM; *n* = 5). The diets (750 g DM/animal per day) were offered in equal amounts at 08:30 and 16:30 h daily for 7 weeks.

### Sample Collection

The animals were slaughtered for sampling after 7 weeks. A segment of the rumen wall from the ventral sac was collected, and the ruminal epithelium was separated from the muscular and serosal layers by blunt dissection and immediately washed three times in ice-cold PBS and frozen immediately in liquid nitrogen. The ruminal epithelium (2 cm × 2 cm) was used for extracting proteins and metabolomics analysis.

### Protein Extraction and Sample Preparation

The total proteins were extracted with RIPA Lysis Buffer (Cat. P0013B, Beyotime Institute of Biotechnology, Shanghai, China). Proteins were dissolved in 50 mM Tris-HCl (pH 8.0) with 8 M urea and incubated for 60 min in a 60°C water bath and alkylated with 1 M iodoacetamide. Subsequently, the samples were incubated for 45 min at room temperature. Finally, proteins on the membrane were dissolved in 50 mM NH_4_HCO_3_ (pH 7.8). The digested protein by trypsin was desalted using a C18 column and then freeze-dried before sample injection.

### Mass Spectrometry

The peptides were first dissolved in buffer A (0.1% formic acid). A 15-cm analytical C18 column (C18, 3 μm, 100 Å) was used for LC separation. The peptides were eluted by a 2–95% gradient of buffer B (aqueous 80% acetonitrile in 0.08% formic acid) at a flow rate of 300 nL/min. The peptides were ionized by nano-electrospray and subsequent tandem mass spectrometry (MS/MS) on a Q Exactive^TM^ Plus (Thermo Fisher Scientific, San Jose, CA, United States) with the electrospray voltage was 2.2 kV. The Orbitrap was performed with full scan MS spectra with a resolution of 60,000 from m/z 350 to 1800.

### Protein Identification and Quantification

The original data was analyzed by Proteome Discoverer (version 1.4, Thermo Fisher Scientific, Waltham, MA, United States). Based on the *Q*-value, we verified the results of protein identification to ensure that the error detection rate was less than 1%. The SIEVE software (Version 2.1 Thermo Scientific, San Jose, CA, United States) was used to analyze two original files for each group by ChromAlign. When alignment scores aligned by retention time and mass is higher than 0.75, it is regarded as a further quantitative analysis. The area under the curve for each group was calculated.

### Metabolite Profiling of the Ruminal Epithelium

The GC-MS analysis has been described previously (Sun et al., 2016). Briefly, 30 mg of the ruminal epithelium was mixed with 900 μL methanol containing ^13^C2-myristic acid (12.5 μg/mL). The mixed liquor were grounded and centrifuged at 20,000 × *g* for 10 min at 4°C. Then, 100 μL of the supernatant was dried in a SpeedVac evaporator (Savant Instruments, Farmingdale, NY, United States). The dried analytes were methoximated with methoxyamine pyridine solution and trimethylsilylated with methyl-trimethyl-silyltrifluoroacetamide.

Thirty microliters of *n*-heptane and methyl stearate (30 μg/mL) were mixed with samples and then the 0.5 μL of mixture was performed by an RTx-5MS column (30 m × 0.25 mm i.d. and film thickness of 0.25 μm; Restek Corporation, Bellefonte, PA, United States). After the raw data were collected, identification of the compounds was achieved by comparison of the mass spectra and retention index of all the detected compounds with authentic reference standards and those available in the National Institute of Standards and Technology Library 2.0.

### Data Analysis

Statistical calculations of metabolomic and proteomic data were carried out by conducting tests using the SPSS software package (SPSS version 18.0.1 for Windows; SPSS Inc., Chicago, IL, United States). The normality of the distribution of the variables was tested using the Shapiro–Wilk test. The independent samples *t*-test procedure was used to analyze the variables found to have a normal distribution. The Kruskal–Wallis test was used to analyze the variables found to have a non-normal distribution. Significance was set at *P* < 0.05.

Principal component analysis (PCA), PLS-DA, and loading plot were carried out using SIMCA-P + 13.0 software (Umetrics, Umeå, Sweden). Variable importance in projection (VIP) was obtained from PLS-DA analysis. Differentially expressed metabolites (VIP > 1.2) and proteins (FC > 1.5) were selected according to VIP and statistical analysis (*P* < 0.05). The differentially expressed metabolites has been analyzed using the MetaboAnalyst web server^1^ for the pathway enrichment analysis.

### Bioinformatic Analysis of Differential Abundance Proteins

The differentially expressed proteins (Table 2) has been analyzed using the OmicsBean^2^ for the protein–protein interaction analysis (PPI) based on gene ontology (GO) enrichment and Kyoto Encyclopedia of Genes and Genomes (KEGG) pathway.

## Results

### Effects of HG Diet Feeding on Ruminal pH and Concentrations of SCVFAs, Lactate, and LPS

It is helpful to briefly describe the data of rumen fermentation published previously (Supplementary Table S1). Briefly, HG feeding decreased the ruminal pH (*P* < 0.001) and increased the concentrations of propionate, butyrate, valerate, isovalerate, total SCVFA, and lactate (*P* < 0.001 to *P* = 0.019).

### Multivariate Analysis of Rumen Tissue Metabolites

**FIGURE 1:**
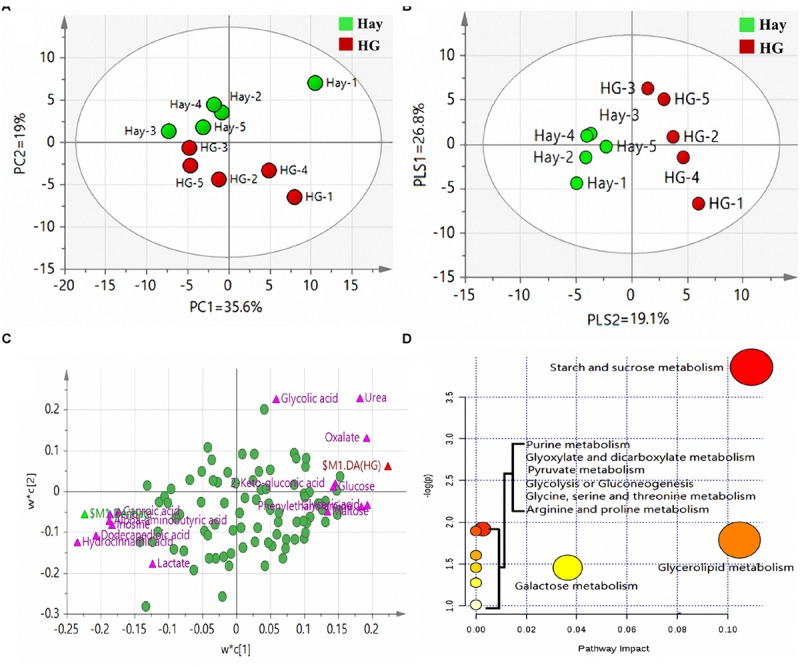
**(A)** Principal component analysis (PCA) of metabolites in rumen epithelium in goats fed hay (Hay) and high grain (HG) diets. PC, the principal component that distinguish the Hay group and the HG group. PC1 is the first principal component; PC2 is the second principal component; PC1 could explained 35.6% of the variation; PC2 explained 19% of the variation. **(B)** Partial least squares discriminant analysis (PLS-DA) of metabolites in rumen epithelium in goats fed hay (Hay) and high grain (HG) diets. PLS, the principal component that distinguish the Hay group and the HG group. PLS1 is the first principal component; PLS2 is the second principal component. **(C)** Loading plot of the 91 commonly detected compounds projected into the PLS-DA model, the most important compounds (VIP > 1.2) responsible for the discrimination are labeled and colored in pink, and the other compounds were colored in green. Compounds are labeled by the names used in Table 1. Variables with the same distance from 0 with similar positions are positively correlated. Those in the opposite direction are negatively correlated. **(D)** Pathway analysis. Plots depicting computed metabolic pathways as a function of –log (*p*) and pathway impact for the key differential metabolites. The impact is the pathway impact value calculated from pathway topology analysis. The larger size indicates higher pathway enrichment, and the darker color indicates higher pathway impact values.

A total of 158 valid peaks were detected by the GC-MS that were unique and non-overlapping in the rumen epithelium samples. After rigorous quality control and identification, we obtained 101 metabolites across all samples. The metabolites mainly included amino acids, carbohydrates, lipids, nucleoside and organic chemicals. PCA was carried out to explore the differences of the metabolites between the two dietary treatments. As shown in Figure 1A, the first two principal components (PCs) can explain 54.6% of the variation. The separation of the two groups in PC 2 revealed significant differences of rumen tissue metabolites in goats fed the HG diet and hay diet, which is particularly apparent in Figure 1B, as analyzed by PLS-DA. PLS-DA scores plots discriminating between the rumen tissue of goats fed Hay and HG diet [predictive ability parameter (*Q*^2^) (cum) = 0.978, goodness-of-fit parameter (*R*^2^) (*Y*) = 0.997]. To investigate the individual rumen tissue metabolites responsible for the variation of the first two PCs, loading plots were used (Figure 1C). The loading plot revealed a statistically significant elevation of glyceric acid, urea, oxalate, 2-keto-gluconic acid, glucose, phenylethanolamine, allonic acid and maltose in the HG group compared with the Hay group. Conversely, the metabolic signature of rumen epithelium tissue in the Hay group consisted of a higher concentration of alpha-aminobutyric acid, caproic acid, inosine, lactate, dodecanedioic acid, hydrocinnamic acid and benzoate. To identify which compounds were responsible for this difference, the following parameters were used as criteria: VIP > 1.2 and *P* < 0.05. As shown in Table 1, eight of the compounds (allonic acid, glyceric acid, glucose, 2-keto-gluconic acid, phenylethanolamine, urea, oxalate, and maltose) were enriched while seven (alpha-aminobutyric acid, caproic acid, inosine, lactate, dodecanedioic acid, hydrocinnamic acid, and benzoate) were reduced in the HG group compared with the Hay group. Through the enrichment analysis, the results showed that starch and sucrose metabolism, purine metabolism, glyoxylate and dicarboxylate metabolism, pyruvate metabolism, glycolysis or gluconeogenesis, glycerolipid metabolism, and galactose metabolism were significantly enriched (*P* < 0.05) with different diets. Through the pathway topology analysis, the results showed that the pathway impact values of 3 metabolic pathways, which included starch and sucrose metabolism, glycerolipid metabolism, and galactose metabolism were higher than 0.03. Based on both the enrichment analysis and impact value, starch and sucrose metabolism, glycerolipid metabolism, and galactose metabolism were closely related with HC diet (Figure 1D).

**Table 1:** Candidate compounds in rumen epithelium that differed between hay (Hay) and high grain (HG) groups.

Compounds	Chemical class	VIP^1^	FC^2^	*P*-value
Alpha-aminobutyric acid	Amino acids	1.86	0.45	0.009
Maltose	Carbohydrates	1.34	6.53	0.047
Allonic acid	Carbohydrates	1.47	2.14	0.028
Glyceric acid	Carbohydrates	1.93	2.11	0.016
Glucose	Carbohydrates	1.43	2.00	0.028
2-Keto-gluconic acid	Carbohydrates	1.45	1.85	0.047
Caproic acid	Lipids	1.72	0.52	0.047
Inosine	Nucleoside	1.82	0.50	0.016
Phenylethanolamine	Organic chemicals	1.84	2.18	0.009
Urea	Organic chemicals	1.82	1.76	0.016
Oxalate	Organic chemicals	1.91	1.70	0.016
Lactate	Organic chemicals	1.23	0.76	0.047
Dodecanedioic acid	Organic chemicals	2.06	0.29	0.009
Hydrocinnamic acid	Organic chemicals	2.32	0.20	0.009
Benzoate	Organic chemicals	1.85	0.50	0.016

*^1^VIP, variable importance in the projection. ^2^FC, fold change, mean value of peak area obtained from HG group/mean value of peak area obtained from Hay group. If the FC value less than 1, it means that metabolites are less in HG group than in Hay group.*

### Identification and Comparison of Proteins of Differential Abundance

Using label-free LC–MS/MS analysis, a total of 2,150 proteins were identified within the false discovery rate of 1%. Following statistical analysis, 35 proteins were found to be differentially expressed in the rumen epithelium between Hay and HG groups, with 12 upregulated and 23 downregulated (Table 2) in the HG group compared with the Hay group. The upregulated proteins were mainly related to oxidative stress, detoxification, immune system processes and anabolism of fatty acid, while the downregulated proteins were mainly related to cell growth and proliferation, protein metabolic processes, and catabolism of fatty acids.

**Table 2:** List of differentially expressed proteins in rumen epithelium from HG group and Hay group.

Protein ID	Gene symbol	Protein name	Ration (HG/Hay)	*P*-value
**Fatty acid metabolism**				
B6UV59	HADHA	Long-chain 3-hydroxyacyl-CoA dehydrogenase	0.38	0.020
W5PGM1	OXCT1	Succinyl-coa:3-ketoacid-coenzyme a transferase	0.01	0.035
K9LQQ8	FABP3	Fatty acid binding protein 3	0.04	0.011
W5QGP1	BDH1	Uncharacterized protein	1.81	0.010
W5Q740	ABCD3	ATP binding cassette subfamily d member 3	0.01	0.044
W5PAS5	HMGCL	Uncharacterized protein	3.03	0.014
**Cell growth and proliferation**				
M4WED3	CDC42	Cell division cycle 42	0.26	0.041
W5PPT6	TUBB	Uncharacterized protein	0.31	0.020
W5QJ02	DHRS7	Uncharacterized protein	1.74	0.024
W5PXP2	SDPR	Uncharacterized protein	0.32	0.032
W5P043	NPM1	Nucleophosmin 1	0.23	0.007
**Carbohydrate metabolic processes**				
D7R7V6	GAPDH	Glyceraldehyde-3-phosphate dehydrogenase	0.56	0.016
W5PK04	PGAM1	Phosphoglycerate mutase family member 1	0.57	0.041
**Oxidative stress and detoxification**				
L0CSP6	PRDX5	Peroxiredoxin-5	2.35	0.038
W5PH14	LOC101117764	UDP-glucuronosyltransferase	2.06	0.037
W5NZJ1	SULT1A1	Sulfotransferase	1.55	0.036
W5PJJ7	LOC101109421	Uncharacterized protein	2.71	0.017
W5P382	ZADH2	Uncharacterized protein	0.02	0.050
W5PHN8	LOC101107119	Uncharacterized protein	3.56	0.014
**Protein metabolic processes**				
W5NS93	PSMA4	Proteasome subunit alpha type	2.39	0.034
W5NSG2	TMEM43	Transmembrane protein 43	0.02	0.025
W5PVT6	UBA1	Ubiquitin-activating enzyme E1	0.60	0.031
W5Q834	ERO1A	Endoplasmic reticulum oxidoreductase 1 alpha	0.55	0.040
W5PAG0	KARS	Lysine–trna ligase	0.02	0.025
W5QB61	FKBP1A	Peptidyl-prolyl *cis*–*trans* isomerase	0.04	0.049
W5Q1N8	RPS23	Small subunit ribosomal protein S23E	0.10	0.025
W5PHW0	HSP90AB1	Heat shock protein 90 alpha family class b member 1	0.59	0.007
**Immune system processes**				
W5Q5H8	FGA	Fibrinogen alpha chain	2.89	0.031
W5PCN2	ANXA7	Annexin	12.29	0.020
**Nucleoside metabolic processes**				
W5PR48	HPRT1	Hypoxanthine phosphoribosyltransferase	0.08	0.035
W5Q3H9	UPP1	Uridine phosphorylase	0.19	0.026
**Ion transport**				
W5PVJ0	N/A	Ferritin	45.13	0.013
W5QGG0	TFRC	Transferrin receptor	0.09	<0.001
W5PK33	EFHD2	Uncharacterized protein	0.43	0.026
W5PP37	ATP5H	Uncharacterized protein	0.18	0.04

### Gene Ontology Annotations of Proteins With Different Abundance

**FIGURE 2:**
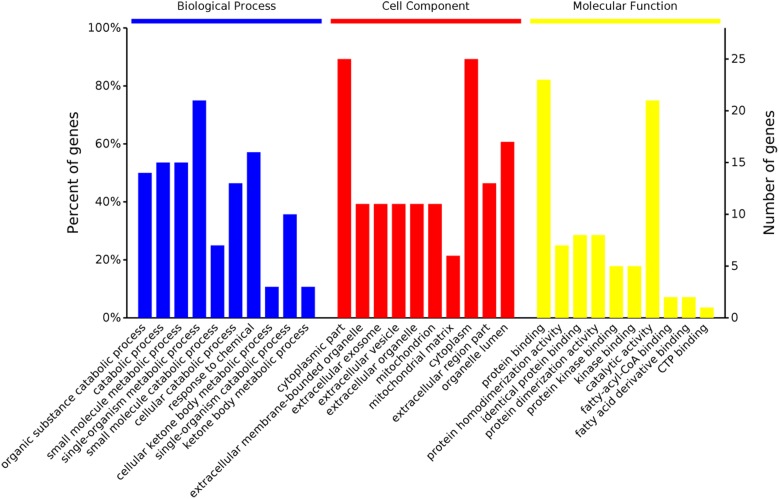
Gene ontology distribution analysis of differentially expressed proteins in the rumen epithelium in goats from the high grain (HG) group and the hay (Hay) group.

In the cellular component group, the differentially expressed proteins were concentrated in the cytoplasmic part and cytoplasm (Figure 2). In the molecular functional group, the differentially expressed proteins that work as binding proteins and catalytic activity were ranked at the top of the category (Figure 2). In the biological process category, the proteins that participate in single-organism metabolic process and response to chemical had the highest ratios among the differentially expressed proteins (Figure 2).

**FIGURE 3:**
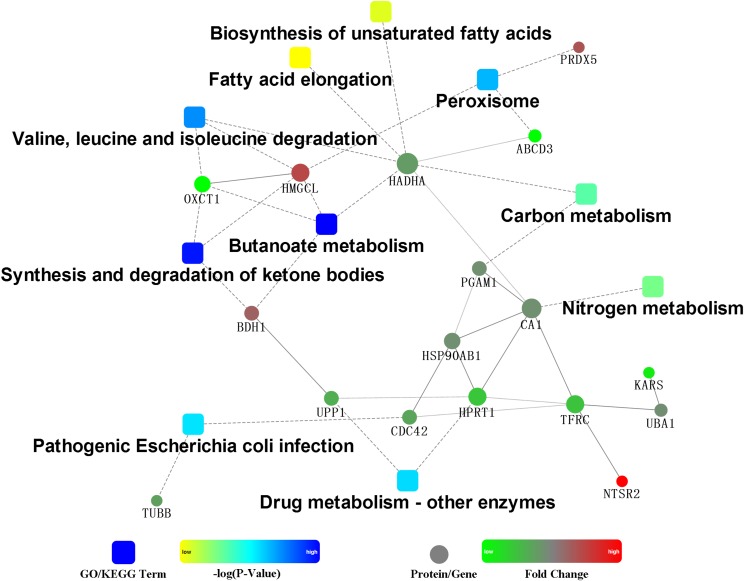
Protein–protein interaction analysis by the OmicsBean.

**FIGURE 4:**
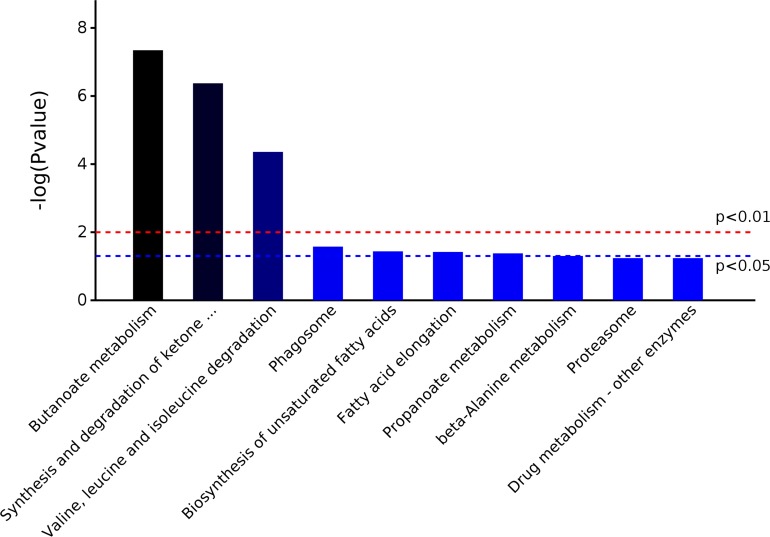
The *p*-value of enriched KEGG pathway.

The PPI of the differentially expressed proteins allow us better understand the key proteins and pathways affected by HG feeding in the rumen epithelium (Figure 3). The PPI network indicated that the protein changes in rumen epithelium were mainly involved in synthesis and degradation of ketone bodies, butanoate metabolism and valine, leucine and isoleucine degradation pathways (Figure 3, 4).

## Discussion

Here, we investigated the relationships among diet, rumen epithelial metabolome and proteome. In the present study, results from PCA and PLS-DA reveal clear differences in the biochemical composition of ruminal tissue of the Hay and the HG fed groups (Figure 1A,B), also demonstrating the evident impact of HG feeding on metabolites of rumen epithelium. This alteration in the composition of compounds in ruminal epithelial tissues may be due to the alternation in the ruminal parameters such as ruminal pH and ruminal metabolites concentration caused by the high grain feeding (Asma et al., 2013).

In the current study, our data revealed that HG diet supports a greater level of maltose in the ruminal epithelial tissues compared with the Hay group (Table 1). As mentioned above, rumen epithelial tissue has many functions including metabolism, nutrient absorption, as well as barrier functions. Normally, maltose cannot be absorbed intact from the lumen of the rumen, however, a decreased ruminal pH, combined with a high LPS level, may impair the ruminal epithelial barrier function (Gäbel et al., 1987; Penner et al., 2011) and further increase the permeability of the epithelium. A possible explanation for this result is that HG feeding resulted in an increase in the permeability of the epithelium. Similarly, a greater abundance of glucose was also detected in the rumen epithelium of goats that were fed an HG diet. In the present study, our data also showed that oxalate level in rumen tissue were significantly greater in HG group (Table 1). A previous study showed that acidification of the caecum in rats enhanced the intestinal oxalate absorption (Diamond et al., 1988). A possible explanation could be the fact that lower luminal pH induced by HG diets may cause more influx of oxalate from the lumen to epithelium.

Hydrocinnamic acid (3-phenylpropionic acid) is derived from the ruminal metabolism of *p*-coumaric and ferulic acids in cellulose (Chesson et al., 1982). Significant down-regulation of hydrocinnamic acid in the HG group was probably due to a lower ruminal cellulose degradation ability than in the Hay group, which is evidenced by the incidence that greater numbers of cellulolytic bacteria existed in hay-fed animals (Fernando et al., 2010). The above findings demonstrated that the cellulose metabolism was affected in rumen of goats fed HG diet. Mao et al. (2016) found that the lower dodecanedioic acid concentrations in rumen fluid in goats fed an HG diet may be caused by acid inhibition of biohydrogenation. Lower levels of dodecanedioic acid in the rumen fluid may result in decreased absorption by the ruminal epithelium. In line with this assumption, in the present study, a significant decrease in dodecanedioic acid level was found in extracts of ruminal epithelium in the HG group (Table 1). Caproic acid, a six-carbon straight-chain fatty acid, is found in trace amounts in rumen fluid. A previous study revealed that a greater ratio of corn starch led to a lower concentration of caproic acid in the rumen (Orskov et al., 1967). The decrease in caproic acid levels in the extracts of ruminal epithelium of the HG group probably can be attributed to the greater ratio of grain in their diet. Phenylethanolamine is a type of biogenic amine detected in the serum of rats and pigs, and it is known to play an important role in mammalian nervous system function (Shannon et al., 1981). Previous studies revealed that HG feeding increased the biogenic amine concentration in rumen fluid of cattle (Wang et al., 2013), thus, in the present study, the increased phenylethanolamine detected in extracts of ruminal epithelium of animal fed HG group may be due to the greater level of phenylethanolamine in rumen fluid of these animals.

Urea is quantitatively the most important end product of nitrogen metabolism in ruminants. Previous studies showed that part of the endogenous urea that is utilized moves into the rumen interior with saliva, but most of it moves from the blood directly through the rumen epithelium (Abdoun et al., 2006). Huntington (1989) reported that feeding a high-starch diet increased the transfer of urea from the blood into the rumen in beef steers, indicating that dietary carbohydrate has a marked effect on urea transfer. Thus, in the present study, a greater urea content in the rumen epithelium in the HG group is reasonable. The current study also revealed that the level of lactate in rumen epithelium tissues in the HG group was lower than that in the Hay group (Table 1). Lactate can be directly absorbed through the rumen wall to the blood and partially converted into ketone bodies in the rumen epithelium (Pennington and Sutherland, 1956). Our results are contrary to a report from a previous study that showed increasing concentrate intake had increased the net portal absorption of lactate in lambs (Huntington et al., 1980). The reason behind this observation is not clear and needs further investigation. Inosine is an endogenous purine nucleoside, which is formed during the breakdown of adenosine by adenosine deaminase (Barankiewicz and Cohen, 1985). The changes in inosine content could indicate a greater amount of metabolic active tissue by a concomitant increased HG intake.

As mentioned earlier, protein expression patterns of the ruminal epithelium of bovine in response to various feeding regimes have been explored using two-dimensional electrophoresis, and differentially expressed proteins that were mainly related to functions involved cellular stress and metabolism (Bondzio et al., 2011). Until now, little information has been available to characterize the goat rumen tissue proteome. In the present study, a total of 34 differentially expressed proteins related to fatty acid metabolism, cell growth and proliferation, carbohydrate metabolic processes, oxidative stress and detoxification, protein metabolic processes, immune system processes, nucleoside metabolic processes and ion transport were detected between the two groups in goats (Table 2). Of these differentially expressed proteins, our data revealed that HG diet feeding decreased the expression of proteins involved in fatty acid metabolism, such as long-chain 3-hydroxyacyl-CoA dehydrogenase (**HADHA**), succinyl-coa:3-ketoacid-coenzyme a transferase (**OXCT1**), and FABP3. Among these downregulated expression of proteins, HADHA protein can catalyze the third step of mitochondrial beta-oxidation (Haglind et al., 2015), FABP3 protein act as a transport of fatty acids to the mitochondria or peroxisome for beta-oxidation (Furuhashi and Hotamisligil, 2008), ATP binding cassette subfamily d member 3 (**ABCD3**) functions as a transporter for moving the fatty acids into peroxisome for beta-oxidation (van Roermund et al., 2014), and OXCT1 was a key enzyme for ketone body utilization (Song et al., 1997). Thus, the downregulated expression of these proteins indicates a decreased catabolism of fatty acids in the HG group. In addition, the present study also revealed that HG diet feeding resulted in a decreased expression of proteins related to the carbohydrate metabolic process, including GAPDH and phosphoglycerate mutase family member 1 (**PGAM1**). It is well known that GAPDH and PGAM1 can catalyze the sixth and eighth step of glycolysis, respectively, and play an important in glucose metabolism. Thus, the reduced expressions of these two proteins in the HG group indicate that HG feeding may result in a decrease in glycolysis in ruminal epithelial tissues.

Our study also demonstrated that seven differentially regulated proteins related to protein metabolism, including lysine–tRNA ligase (**KARS**), endoplasmic reticulum oxidoreductase 1 alpha (**ERO1A**), proteasome subunit alpha type (**PSMA4**), peptidyl-prolyl *cis*–*trans* isomerase (**FKBP1A**), small subunit ribosomal protein S23E (**RPS23**), HSP90AB1, and UBA1, were affected by HG feeing in goats. Of these proteins mentioned earlier, ERO1A is reported to be involved in the essential step of correct protein folding for the formation of disulfide bonds by reoxidizing protein disulfide isomerase (Gess et al., 2003). In the present study, the downregulation of ERO1A might relate to the decrease in the protein disulfide-isomerase, as Hollmann et al. (2013) reported that HG feeding decreased the protein disulfide-isomerase which plays a synergistic effect on protein folding with ERO1A (Santos et al., 2009). Regarding other differentially regulated proteins involved in protein metabolism, RPS23 is reported to be involved in protein synthesis, while KARS plays an important role in the process of inserting lysine into proteins (Freist and Gauss, 1995). FKBP1A is believed to play an important role in accelerating the rate at which proteins fold into their native conformation (Siekierka et al., 1990), and PSMA4 and UBA1 are involved in the ubiquitin-proteasome proteolytic pathway (Ciechanover, 1994). HSP90AB1 plays a key role in protein folding, degradation, and morphological evolution. Generally, the findings of these differentially regulated proteins suggest that HG feeding affected the synthesis, correct folding, and breaking down of proteins in rumen epithelium.

Interestingly, in the present study, six proteins, PRDX5, UDP-glucuronosyltransferase (LOC101117764), sulfotransferase (SULT1A1), ZADH2, LOC101107119, and LOC101109421, related to oxidative stress and detoxification were upregulated in response to an HG diet. Of these upregulated expression of protein, SULT1A1 and LOC101117764 are key components of the body’s chemical defense system and these two enzymes protein are believed to play an important role in maintaining host health (Rubin et al., 1996; Xu et al., 2005). In the present study, the increase in the relative expression of these two enzymes protein probably indicates the HG feeding may result in an enhancement in host deference in response to more toxic substances such as endotoxin and biogenic amine translocating from the rumen into the rumen epithelium (Eisenhofer et al., 1997). In the present study, we also found HG feeding upregulated immune system processes-related ANXA7 proteins, and this is consistent with the report by Bondzio et al. (2011) who found increased expression of ANXA7 in the rumen epithelium in response to HG diets. As ANXA7 is one of the annexin family members that is upregulated in inflammatory myopathies (Probst-Cousin et al., 2004), thus, the upregulation of ANXA7 imply that HG feeding may trigger an inflammatory response in rumen epithelium, and this peculations corresponds well to the findings in our previous report that HG feeding caused local inflammation of the rumen epithelium (Liu et al., 2013).

Several proteins related to ion transportation were also different between the two groups. Transferrin receptor (**TFRC**) is present on the surface of cells and binds to transferrin to transport iron into the cell. A previous work showed decreased TFRC combined with increased ferritin, which may indicate a disorder of iron metabolism in the HG group. In line with our findings, Hollmann et al. (2013) found downregulation of transferrin in the rumen epithelium in response to HG diets. The previous study has already reported that HG diets decrease the duration time of the cell cycle in the ruminal epithelium, and the rates of cell division and turnover are considered to be related to the pathological conditions, including hyperkeratosis, parakeratosis, and ruminitis (Goodlad, 1981). In the present study, six differentially regulated proteins related to cell growth and proliferation were observed. Given that the inhibition of CDC42 and Nucleophosmin 1 (**NPM1**) could induce cell apoptosis (Ambrogio et al., 2008), the downregulation of them in response to HG diets may be attributed to accelerated turnover by promoting cell apoptosis in the ruminal epithelium.

PPI showed that three pathways were enriched in the KEGG, including butanoate metabolism, synthesis and degradation of ketone bodies, and valine, leucine and isoleucine degradation. Although it was well established that rumen epithelium plays a crucial role in SCVFA absorption and metabolism (Bannink et al., 2016), no other differentially expressed protein related to other SCVFAs uptake and metabolism was detected except for the protein related to butyrate metabolism. This may be because butyrate is more readily absorbed and metabolized in the rumen epithelium than other SCVFAs (Pennington, 1952). Interestingly, in line with our research, only gene expression related to butyrate metabolism was upregulated in lower risk of SARA cows compared to higher risk of SARA cows (Gao and Oba, 2016), indicating butyrate metabolism in the rumen epithelium could be more crucial for SCVFA metabolism and rumen epithelial function.

## Conclusion

In general, our data showed that long-term feeding of an HG diet discriminatively altered the protein expression (with 12 upregulated and 23 downregulated proteins) and metabolites profiles (with 8 increased metabolites and 7 decreased metabolites). The downregulated proteins were related to fatty acid metabolism, carbohydrate metabolic processes and nucleoside metabolic processes, while most of upregulated proteins were related to oxidative stress and detoxification. The enrichment analysis of different metabolites indicated that HG diet mainly affected starch and sucrose metabolism, purine metabolism, glyoxylate and dicarboxylate metabolism, glycerolipid metabolism, pyruvate metabolism, glycolysis or gluconeogenesis, galactose metabolism, glycine, serine and threonine metabolism, and arginine and proline metabolism. These findings may contribute to better understanding how rumen epithelia adapt to HG feeding.

## Author Contributions

CG and DS carried out the majority of the animal studies. CG and SM carried out data interpretation and manuscript preparation. SM and XW were responsible for the conception of the project and the oversight of the experiments.

## Conflict of Interest Statement

The authors declare that the research was conducted in the absence of any commercial or financial relationships that could be construed as a potential conflict of interest.

## Footnotes

1 http://www.metaboanalyst.ca

2 http://www.omicsbean.cn/
