# Supplementary material for: A Combined Metabolomic and Proteomic Study Revealed the Difference in Metabolite and Protein Expression Profiles in Ruminal Tissue From Goats Fed Hay or High-Grain Diets
Source: Front Physiol. 2019 Feb 8;10:66. doi: 10.3389/fphys.2019.00066 (PMC6375843; doi:10.3389/fphys.2019.00066)
Supplement: Supplementary file 1 [file Table_1.DOCX]

**Table S1** The effect of feeding hay (Hay) or high-grain (HG) diet on rumen fermentation in goats at the time of slaughter^1^

| Item | Hay | HG | *P*-value |
| --- | --- | --- | --- |
| pH | 6.12 ± 0.09 | 5.33 ± 0.09 | <0.001 |
| Total VFA, mM | 93.90 ± 1.25 | 116.16 ± 4.25 | 0.001 |
| Acetate, mM | 70.24 ± 0.88 | 63.12 ± 1.89 | 0.009 |
| Propionate, mM | 14.57 ± 0.29 | 34.12 ± 2.24 | <0.001 |
| Butyrate, mM | 7.15 ± 0.39 | 15.81 ± 0.94 | <0.001 |
| Isobutyrate, mM | 1.09 ± 0.10 | 1.43 ± 0.15 | 0.089 |
| Valerate, mM | 0.45 ± 0.04 | 0.74 ± 0.05 | 0.002 |
| Isovalerate, mM | 0.41 ± 0.12 | 0.94 ± 0.14 | 0.019 |
| Acetate: Propionate | 4.82 ± 0.05 | 1.88 ± 0.10 | <0.001 |
| Lactic acid, mM | 0.26 ± 0.02 | 0.67 ± 0.04 | <0.001 |
| Free LPS, EU/mL^2^ | 22,547 ± 1,065 | 64,628 ± 1,685 | <0.001 |

^1^Data are presented as mean ± Standard Error of Mean.

^2^LPS, Lipopolysaccharides; EU, endotoxin unit
